# Gender bias in clinical trials of biological agents for severe asthma: A systematic review

**DOI:** 10.1371/journal.pone.0257765

**Published:** 2021-09-23

**Authors:** Pablo Ciudad-Gutiérrez, Beatriz Fernández-Rubio, Ana Belén Guisado-Gil

**Affiliations:** Servicio de Farmacia, Hospital Universitario Virgen del Rocío, Sevilla, Spain; Humanitas University, ITALY

## Abstract

Asthma is one of the most common chronic diseases characterized by sex disparities. Gender bias is a well-documented issue detected in the design of published clinical trials (CTs). International guidelines encourage researchers to analyze clinical data by sex, gender, or both where appropriate. The objective of this work was to evaluate gender bias in the published CTs of biological agents for the treatment of severe asthma. A systematic review of randomized controlled CTs of the biological agents (omalizumab, benralizumab, reslizumab, mepolizumab or dupilumab) for the treatment of severe asthma was conducted. The literature search was performed using PubMed and EMBASE without language restrictions. This study followed the corresponding international recommendations. We identified a total of 426 articles, of which 37 were finally included. Women represented 60.4% of patients included. The mean percentage of women in these trials was 59.9%, ranged from 40.8% to 76.7%. The separate analysis by sex of the main variable was only performed in 5 of the 37 publications included, and none of the trials analyzed secondary variables by sex. Only 1 of the articles discussed the results separately by sex. No study included the concept of gender in the text or analyzed the results separately by gender. The proportion of women included in CTs was higher compared to publications of other disciplines, where women were under-represented. The analysis of the main and secondary variables by sex or gender, even the discussion separately by sex, was insufficient. This gives rise to potential gender bias in these CTs.

## 1. Introduction

The term “gender” in research could be defined as a systematic mistake associated with social construct, which incorrectly regards women and men as similar/different [1], whereas the term “sex” is related to biological characteristics based upon chromosomal assignment [2]. In 1993, The Food and Drug Administration (FDA) [3] published a guideline regarding the participation of women in clinical trials (CTs) and evaluating all clinical data by sex, but it was not reproduced in Europe [4]. A short time ago, The Sex and Gender Equity in Research (SAGER) guideline proposed analyzing clinical data by sex, gender, or both where appropriate [5].

Gender bias is a well-established term used in biomedical research to show the low sensitivity to gender among low women representation and absence of analysis separately by sex in CTs [6–9]. In Psychiatry, three studies about gender bias remarked that results were poorly stratified by sex [10–12]. In Neurology, one study conducted in 2015 highlighted that women were only represented in 19% of CTs included [13] and a systematic review in multiple sclerosis pointed out that only 15 of 55 studies included an analysis by sex of the primary endpoint [14]. Additionally, one article about gender bias in pulmonary diseases found out an under-diagnosis of chronic pulmonary obstructive disease in women (42%) with respect to men (58%) because higher smoking rates are usually attributed to the male population [15].

Asthma is one of the most common chronic and non-communicable diseases that affects around 334 million people worldwide, and its prevalence has been increasing by 50% every decade [16]. Many epidemiologic studies mention the presence of sex disparities in asthma prevalence and severity [17, 18]. As children, boys have an increased prevalence of asthma compared to girls (11.9% vs. 7.5%, respectively), and boys are also twice as likely as girls to be hospitalized for an asthma exacerbation [19]. However, during adolescence, there is a decline in asthma prevalence and morbidity in males concurrent with an increase in females. By adulthood, women have increased asthma prevalence compared to men (9.6% versus 6.3%, respectively), and women are three times more likely than men to be hospitalized for an asthma-related event [20].

Approximately 5–10% of asthmatic patients experience “severe asthma” because they require treatment with high-dose inhaled corticosteroids plus a long-acting beta-adrenoceptor agonist (LABA), leukotriene modifier or theophylline and/or systemic corticosteroids as background therapy to prevent a poor asthma control [21]. Over the past decade, an improved understanding of the complex pathophysiology of asthma has led to the development of new classification for phenotypes of asthma. This classification is based on clinical, physiological and inflammatory parameters in order to assign asthmatic patients to “phenotypic clusters”. Biological agents have demonstrated a beneficial role in certain clusters targeted at immunoglobulin E (IgE) or eosinophils [22]. Omalizumab was the first monoclonal antibody (mAb) developed for a specific subgroup of patients with uncontrolled IgE-mediated allergic asthma. Recently, anti-interleukin-5 (IL-5) (benralizumab, reslizumab and mepolizumab) and also anti-IL-4/IL-13 drugs (dupilumab) are been approved for patients with uncontrolled eosinophilic asthma [23].

Currently, the biological treatment with monoclonal antibodies has been shown to reduce asthma exacerbations and oral corticosteroid use, and improve lung function and quality of life in appropriately selected patients [24]. Because of differences in gender, analysis of gender bias should be taken into consideration when evaluating the efficacy and safety of asthma novel therapies [25]. Thus, this systematic review aims to evaluate gender bias in published CTs of omalizumab, benralizumab, reslizumab, mepolizumab and dupilumab in severe asthma.

## 2. Materials and methods

### 2.1 Eligibility criteria

This review protocol was registered on the International Platform of Registered Systematic Review and Meta-analysis Protocols (INPLASY, DOI number: 10.37766/inplasy2021.1.0020) and is reported according to the Preferred Reporting Items for Systematic Reviews and Meta-Analyses (PRISMA) Equity 2012 Extension declaration [26]. We selected the studies that met the following inclusion criteria:

- The study drug was omalizumab, benralizumab, reslizumab, mepolizumab or dupilumab.
- CTs with a control group and random assignment.
- Patients treated could be pediatrics or adults.
- The aim of the CTs was the evaluation of the efficacy and safety of the study drug. CTs that additionally assessed other variables such as quality of life or pharmacokinetic/pharmacodynamics were not excluded.
- Patients were diagnosed with severe asthma, with an eosinophilic or allergic phenotype.

We excluded:

- CTs in phase I.
- Post-hoc analysis of one or several previously published CTs, extension CTs of previously published trials as well as systematic reviews and meta-analysis. These works included the same patients that were evaluated in their original articles.
- Pilot studies with a small sample of patients (n<50), short reports and letters to the editor, due to the absence of complete data from larger CTs.
- CTs that involved the evaluation of the treatment regimens based on mAb plus other therapies. Those studies that allowed concomitant medications, that is, drugs that are not being studied but which a patient is taking through all or part of a study, were included.

### 2.2 Information sources

An electronic literature search was performed using PubMed and EMBASE on May 1 2020, with no publication date or language restrictions. Search terms included a mixture of MeSH terms and free text (keywords and synonyms) combined with Boolean operators. The search strategy is detailed in Table 1. Besides, the reference lists of selected studies were hand-searched to identify any other relevant studies.

**Table 1:** Complete search strategy for different databases.

Healthcare Database	Search strategy
**PubMed**	(omalizumab)AND ((severe asthma) OR (allergic asthma) OR (eosinophilic asthma) OR (refractory asthma)) AND (randomizedcontrolledtrial[Filter])
	(reslizumab)AND ((severe asthma) OR (allergic asthma) OR (eosinophilic asthma) OR (refractory asthma)) AND (randomizedcontrolledtrial[Filter])
	(mepolizumab)AND ((severe asthma) OR (allergic asthma) OR (eosinophilic asthma) OR (refractory asthma)) AND (randomizedcontrolledtrial[Filter])
	(dupilumab)AND ((severe asthma) OR (allergic asthma) OR (eosinophilic asthma) OR (refractory asthma)) AND (randomizedcontrolledtrial[Filter])
	(benralizumab)AND ((severe asthma) OR (allergic asthma) OR (eosinophilic asthma) OR (refractory asthma)) AND (randomizedcontrolledtrial[Filter])
**EMBASE**	omalizumab:ab,ti AND (’severe asthma’:ab,ti OR ’allergic asthma’:ab,ti OR ’eosinophilic asthma’:ab,ti OR ’refractory asthma’:ab,ti) AND [randomized controlled trial]/lim
	reslizumab:ab,ti AND (’severe asthma’:ab,ti OR ’allergic asthma’:ab,ti OR ’eosinophilic asthma’:ab,ti OR ’refractory asthma’:ab,ti) AND [randomized controlled trial]/lim
	mepolizumab:ab,ti AND (’severe asthma’:ab,ti OR ’allergic asthma’:ab,ti OR ’eosinophilic asthma’:ab,ti OR ’refractory asthma’:ab,ti) AND [randomized controlled trial]/lim
	dupilumab:ab,ti AND (’severe asthma’:ab,ti OR ’allergic asthma’:ab,ti OR ’eosinophilic asthma’:ab,ti OR
	’refractory asthma’:ab,ti) AND [randomized controlled trial]/lim
	benralizumab:ab,ti AND (’severe asthma’:ab,ti OR ’allergic asthma’:ab,ti OR ’eosinophilic asthma’:ab,ti OR ’refractory asthma’:ab,ti) AND [randomized controlled trial]/lim

### 2.3 Study selection

Two independent reviewers (BFR and PCG) screened the titles and abstracts of all eligible publications for possible inclusion. To ensure inter-rater reliability, 100% of the articles were assessed independently by both authors. The articles included were full-length read before a final decision on inclusion. Any disagreement was settled by consensus with a third reviewer (ABGG).

### 2.4 Data collection and analysis

Reviewers independently extracted data and ABGG examined all extraction sheets to ensure their accuracy. We explicitly stated if there were any missing data from CTs. For each publication, the following variables were registered:

- Drug in research: omalizumab, benralizumab, reslizumab, mepolizumab or dupilumab.
- Year of publication.
- Age: 6 to 11 years (pediatrics patients) and/or ≥12 years old (adult patients).
- Financing of the trial: pharmaceutical industry or independent (the CTs were considered to be promoted by pharmaceutical companies if one of the authors was employed by a pharmaceutical company or if direct funding was specified).
- Location: United States, Europe, Japan, Asia, Australia or the rest of the world (ROW).
- Trial phase: I, IIa, IIb, IIIa, IIIb or IV.
- Comparator: placebo or active drug (best standard of care or optimized asthma therapy).
- Objectives of the trial: efficacy and safety, and if a pharmacodynamic/pharmacokinetic evaluation was performed.
- Diagnosis: severe allergic, referring to the one that requires treatment with high dose inhaled corticosteroids plus a long-acting beta-adrenoceptor agonist (LABA), leukotriene modifier or theophylline and/or systemic corticosteroids, or uncontrolled eosinophilic asthma, caused by high levels of eosinophils.
- Asthma controllers at entry: medication that patients were taking for asthma control before starting the clinical trial (inhaled corticosteroids, long-acting beta-agonists, leukotriene receptor antagonists, oral corticosteroids, or short-acting beta-agonists)

For the analysis of gender and sex differences and in order to characterize the gender sensitivity of the trials, we followed the Spanish recommendations for the study and evaluation of gender differences in CTs of drugs [27], the FDA guide [3] and the European Commission [28]. In the same way, the methodology was based on the SAGER guidelines [5] similar recommendations published in Canada [29] and previous publications [11]. The variables analyzed were:

- Percentage of female authors among all authors.
- The number of patients recruited.
- The number of women included and the percentage of women among patients recruited.
- If there were (or not) sex or gender-stratified results of the main and secondary outcomes.
- If the discussion of the results was analyzed by sex and gender.
- If pregnancy was cited as an exclusion criteria, the studies analyzed the interaction between hormone replacement therapy and study drug, included women using hormonal contraceptives, analyzed the interaction between hormonal contraceptives and the study drug, analyzed the influence of the drug on the pharmacokinetics of hormonal contraceptives, investigated the effects of the phase of the menstrual cycle on the response to the drug, and studied the influence of the phase of the menstrual cycle on the pharmacokinetics of the drug.

We also applied a subgroup analysis for the variables: date of publication, location, comparator, drug, age of patients, objectives and sample size.

## 3. Results

**Fig 1:**
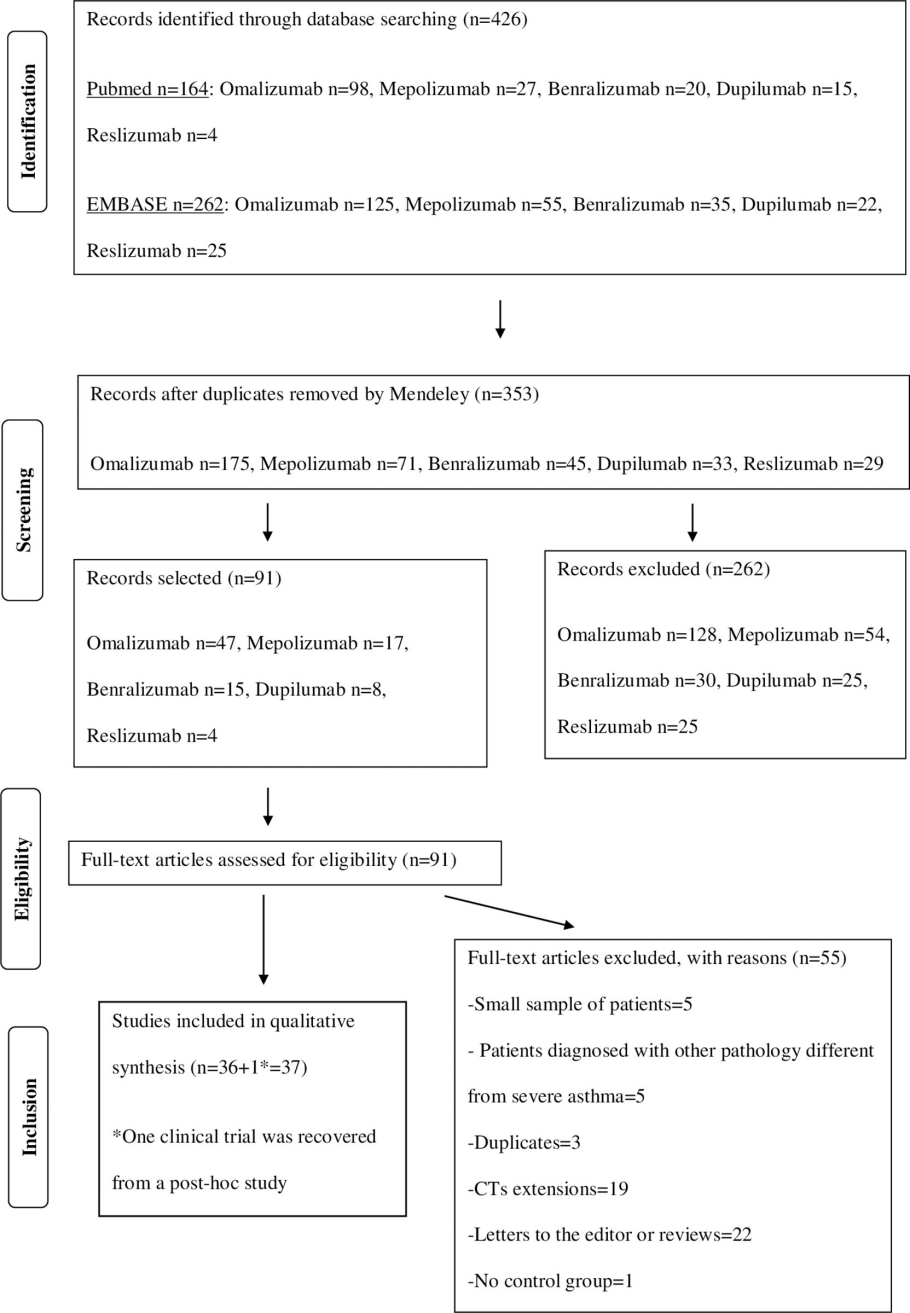
Study selection flowchart.

426 records were identified through database searching. After the elimination of duplicates, 353 records were screened by title and abstract. We assessed 91 articles for eligibility; 55 were excluded because they did not meet the eligibility criteria. One clinical trial [30] was identified from a post-hoc study, so 37 studies were finally included Fig 1.

Table 2 indicates the characteristics of the trials included in the study [30–66]. In most publications, the study drug was omalizumab (16), followed by benralizumab (9), mepolizumab (5), dupilumab (4) and reslizumab (3). The age of patients was ≥12 in 34 studies, <12 in two studies and only one included a population ranging from 6 to 20 years. Most trials were funded by pharmaceutical companies. The majority of the studies were carried out worldwide (25), followed by those accomplished in the United States (7), Europe (2), Asia (1), Japan (1) and the United States + Canada (1). Twenty-one trials were in phase III, 8 in phase II, 4 in phase IV and the rest were not specified (4). The comparator was placebo in 35 trials and other asthma therapies (“best standard care or optimized asthma therapy”) in the remaining studies. The trials measured the variables of efficacy and safety (35), efficacy, safety and quality of life (1) and efficacy, safety and pharmacokinetic (1). Sixteen studies included patients with severe allergic asthma and 21 with uncontrolled eosinophilic asthma. Most patients were treated with inhaled corticosteroids + LABA as asthma controllers before starting biological treatment.

**Table 2:** Characteristics of CTs included.

Study	Drug	Age of patients (years)	Funding	Location	Phase	Comparator	Objectives	Diagnosis	Asthma controllers at entry
Busse et al., 2001	Omalizumab	≥12	Ph. companies	Worldwide	III	Placebo	Efficacy and safety	SAA	ICs + SABA
Milgrom et al., 2001	Omalizumab	<12	Ph. companies	USA	III	Placebo	Efficacy and safety	SAA	ICs + SABA
Soler et al., 2001	Omalizumab	≥12	Ph. companies	Worldwide	II	Placebo	Efficacy and safety	SAA	ICs + SABA
Ayres et al., 2004	Omalizumab	≥12	Ph. companies	Europa	-	Best standard of care	Efficacy and safety	SAA	ICs + SABA
Holgate et al., 2004	Omalizumab	≥12	Ph. companies	Worldwide	III	Placebo	Efficacy and safety	SAA	ICs + SABA + LABA
Vignola et al., 2004	Omalizumab	≥12	Ph. companies	Worldwide	-	Placebo	Efficacy and safety	SAA	ICs + SABA
Humbert et al., 2005	Omalizumab	≥12	None	Worldwide	III	Placebo	Efficacy and safety	SAA	ICs + LABA
Lanier et al., 2009	Omalizumab	<12	Ph. companies	USA	III	Placebo	Efficacy and safety	SAA	ICs+ SABA
Ohta et al., 2009	Omalizumab	≥12	Ph. companies	Japón	III	Placebo	Efficacy and safety	SAA	ICs +LABA, LTRAs, OCs and theophylline
Bousquet et al., 2011	Omalizumab	≥12	Ph. companies	Worldwide	IV	OAT	Efficacy and safety	SAA	ICs + LABA
Busse et al., 2011	Omalizumab	6–20	Ph. companies	USA	IV	Placebo	Efficacy and safety	SAA	ICs + LABA
Hanania et al., 2011	Omalizumab	≥12	Ph. companies	Worldwide	IIIb	Placebo	Efficacy and safety	SAA	ICs + LABA
Bardelas et al., 2012	Omalizumab	≥12	Ph. companies	USA	IV	Placebo	Efficacy and safety	SAA	ICs + LABA, LTRAs, theophylline and zileuton
Rubin et al., 2012	Omalizumab	≥12	Ph. companies	Worldwide	III	Placebo	Efficacy, safety and QL	SAA	ICs + LABA
Li et al., 2016	Omalizumab	≥12	Ph. companies	Worldwide	III	Placebo	Efficacy and safety	SAA	ICs + LABA
Ledford et al., 2017	Omalizumab	≥12	Ph. companies	USA	IV	Placebo	Efficacy and safety	SAA	ICs + LABA
Castro et al., 2014	Benralizumab	≥12	Ph. companies	Worldwide	IIb	Placebo	Efficacy and safety	SEA	ICs + LABA
Nowak et al., 2015	Benralizumab	≥12	Ph. companies	USA + Canada	II	Placebo	Efficacy and safety	SEA	ICs + LABA
Bleecker et al., 2016	Benralizumab	≥12	Ph. companies	Worldwide	III	Placebo	Efficacy and safety	SEA	ICs + LABA
Fitzgerald et al., 2016	Benralizumab	≥12	Ph. companies	Worldwide	III	Placebo	Efficacy and safety	SEA	ICs + LABA
Park et al., 2016	Benralizumab	≥12	Ph. companies	Asia	IIa	Placebo	Efficacy and safety	SEA	ICs + LABA
Ferguson et al., 2017	Benralizumab	≥12	Ph. companies	Worldwide	III	Placebo	Efficacy and safety	SEA	ICs + LABA
Nair et al., 2017	Benralizumab	≥12	Ph. companies	Worldwide	III	Placebo	Efficacy and safety	SEA	ICs + LABA
Zeitlin et al., 2018	Benralizumab	≥12	Ph. companies	USA	IIIb	Placebo	Efficacy, safety and PK	SEA	ICs + LABA
Panettieri et al., 2020	Benralizumab	≥12	Ph. companies	Worldwide	III	Placebo	Efficacy and safety	SEA	ICs + LABA
Flood-Page et al., 2007	Mepolizumab	≥12	Ph. companies	Worldwide	II	Placebo	Efficacy and safety	SEA	ICs+ SABA
Haldar et al., 2009	Mepolizumab	≥12	Ph. companies	Europa	-	Placebo	Efficacy and safety	SEA	OCs+ SABA
Pavord et al., 2012	Mepolizumab	≥12	Ph. companies	Worldwide	II	Placebo	Efficacy and safety	SEA	OCs+ SABA
Bel et al., 2014	Mepolizumab	≥12	Ph. companies	Worldwide	III	Placebo	Efficacy and safety	SEA	OCs + ICs
Ortega et al., 2014	Mepolizumab	≥12	Ph. companies	Worldwide	III	Placebo	Efficacy and safety	SEA	ICs + OCs
Wenzel et al., 2013	Dupilumab	≥12	Ph. companies	USA	IIa	Placebo	Efficacy and safety	SEA	ICs + LABA
Wenzel et al., 2016	Dupilumab	≥12	Ph. companies	Worldwide	IIb	Placebo	Efficacy and safety	SEA	ICs + LABA
Castro et al., 2018	Dupilumab	≥12	Ph. companies	Worldwide	III	Placebo	Efficacy and safety	SEA	ICs + LABA+ LTRAs
Rabe et al., 2018	Dupilumab	≥12	Ph. companies	Worldwide	III	Placebo	Efficacy and safety	SEA	ICs + OCs + LABA+ LTRAs
Castro et al., 2011	Reslizumab	≥12	Ph. companies	Worldwide	-	Placebo	Efficacy and safety	SEA	ICs + LABA + LTRAs and sodium cromoglycate
Castro et al., 2015	Reslizumab	≥12	Ph. companies	Worldwide	III	Placebo	Efficacy and safety	SEA	ICs+ LABA, LTRAs and cromolyn sodium
Bjermer et al., 2016	Reslizumab	≥12	Ph. companies	Worldwide	III	Placebo	Efficacy and safety	SEA	ICs+ LABA, SABA, LTRAs and cromolynsodium

Abbreviations: ICs = inhaled corticosteroids, LABA = long-acting beta-agonists, LTRAs = leukotriene receptor antagonists, OAT = Optimized Asthma Therapy, OCs = oral corticosteroids, Ph. Companies = Pharmaceutical companies, PK = Pharmacokinetic, QL = Quality of Life, SAA = Severe Allergic Asthma, SABA = short-acting beta-agonists, SEA = Severe Eosinophilic Asthma, USA = United States of America.

Table 3 shows the sex-related characteristics of the studies. The mean percentage of female authors among all the authors was 17.5% (range 0–37.5). The total number of patients included in these studies was 16742. The average number of patients per study was 452 (range 61–1902). There were 10108 participants women, with an average number of women per study of 273 (range 29–1197). Women represented 60.4% of patients included. The mean percentage of women in these trials was 59.9%, ranged from 40.8% to 76.7%. The separate analysis by sex of the main variable was carried out in only 5 of the 37 studies included. Moreover, none of the studies analyzed secondary variables between the subpopulation of men and women. Only 1 of the 37 trials discussed results separated by sex. No study included the concept of gender in the text or analyzed the results separately by gender. Pregnancy was an exclusion criterion in 11 trials. None of the included studies analyzed any of the other gender or sex-related variables.

**Table 3:** Proportion of women and other characteristics of sex assessment.

Study	Total of patients	Total of women	Percentage of women	Analysis by sex of the main outcome	Analysis by sex of secondary outcomes	Discussed results analyzed by sex
Busse et al., 2001	525	310	59.0%	No	No	No
Milgrom et al., 2001	334	231	69.2%	No	No	No
Soler et al., 2001	546	278	50.9%	No	No	No
Ayres et al., 2004	312	220	70.5%	No	No	No
Holgate et al., 2004	246	150	61.0%	No	No	No
Vignola et al., 2004	405	223	55.1%	No	No	No
Humbert et al., 2005	419	279	66.6%	No	No	No
Lanier et al., 2009	628	203	32.3%	No	No	No
Ohta et al., 2009	315	171	54.3%	No	No	No
Bousquet et al., 2011	400	259	64.8%	No	No	No
Busse et al., 2011	419	177	42.2%	No	No	No
Hanania et al., 2011	850	557	65.5%	No	No	No
Bardelas et al., 2012	271	180	66.4%	No	No	No
Rubin et al., 2012	116	89	76.7%	No	No	No
Li et al., 2016	609	328	53.9%	Yes	No	No
Ledford et al., 2017	176	123	69.9%	Yes	No	No
Castro et al., 2014	606	417	68.8%	No	No	No
Nowak et al., 2015	110	77	70.0%	No	No	Yes
Bleecker et al., 2016	1205	796	66.1%	No	No	No
Fitzgerald et al., 2016	1306	807	61.8%	No	No	No
Park et al., 2016	106	65	61.3%	No	No	No
Ferguson et al., 2017	211	129	61.1%	Yes	No	No
Nair et al., 2017	220	135	61.4%	No	No	No
Zeitlin et al., 2018	103	42	40.8%	No	No	No
Panettieri et al., 2020	233	157	67.4%	Yes	No	No
Flood-Page et al., 2007	362	202	55.8%	No	No	No
Haldar et al., 2009	61	29	47.5%	No	No	No
Pavord et al., 2012	621	387	62.3%	No	No	No
Bel et al., 2014	135	74	54.8%	No	No	No
Ortega et al., 2014	576	329	57.1%	No	No	No
Wenzel et al., 2013	104	52	50.0%	No	No	No
Wenzel et al., 2016	776	490	63.1%	No	No	No
Castro et al., 2018	1902	1197	62.9%	No	No	No
Rabe et al., 2018	210	127	60.5%	No	No	No
Castro et al., 2011	106	63	59.4%	No	No	No
Castro et al., 2015	953	581	61.0%	No	No	No
Bjermer et al., 2016	265	174	65.7%	Yes	No	No
**Total**	**16742**	**10108**	**60.4**%	**5/37**	**0/37**	**1/37**

Table 4 shows the proportion of women and sex-related characteristics in the different subgroups of the CTs. The five trials that considered the analysis by sex in the main outcome were carried out with patients ≥12 years using placebo as comparator. Moreover, they were published between 2011 and 2020 and aimed at efficacy and safety evaluation.

**Table 4:** Proportion of women and other characteristics of sex assessment according to the different subgroups.

	Studies	Representation of women			Analysis by sex		
Subgroup	N	N patients	N women	Percentage	Analysis by sex of the main outcome	Analysis by sex of secondary outcomes	Discussion of results by sex
					N/N Total Studies	N/N Total Studies	N/N Total Studies
Total	37	16742	10108	60.4%	5/37	0/37	1/37
Geography							
USA	7	2035	1008	49.5%	1/37	0/37	0/37
USA+Canada	1	110	77	70.0%	0/37	0/37	1/37
EU	2	373	249	66.8%	0/37	0/37	0/37
Global	25	13803	8538	61.9%	4/37	0/37	0/37
Asia/Japan	2	421	236	56.1%	0/37	0/37	0/37
Drugs in study							
Benralizumab	9	4100	2625	64.0%	2/37	0/37	1/37
Dupilumab	4	2992	1866	62.4%	0/37	0/37	0/37
Mepolizumab	5	1755	1021	58.2%	0/37	0/37	0/37
Omalizumab	16	6571	3778	57.5%	3/37	0/37	0/37
Reslizumab	3	1324	818	61.8%	0/37	0/37	0/37
Age of patients							
< 12 years	2	962	432	44.9%	0/37	0/37	0/37
≥ 12 years	34	15361	9559	62.2%	5/37	0/37	1/37
6–20 years	1	419	117	27.9%	0/37	0/37	0/37
Comparator							
Placebo	35	16030	9629	60.1%	5/37	0/37	1/37
BSC	1	312	220	70.5%	0/37	0/37	0/37
OAT	1	400	259	64.8%	0/37	0/37	0/37
Date of publication							
2001–2010	11	4153	2296	55.3%	0/37	0/37	0/37
2011–2020	26	12589	7812	62.1%	5/37	0/37	1/37
Outcome							
Efficacy+Safety	35	16523	9977	60.4%	5/37	0/37	1/37
Efficacy+Safety+PK	1	103	42	40.8%	0/37	0/37	0/37
Efficacy+Safety+QL	1	116	89	76.7%	0/37	0/37	0/37
Sample size							
N 0–100	1	61	29	47.5%	0/37	0/37	0/37
N 101–500	23	5578	3399	60.9%	4/37	0/37	1/37
N 501–1000	10	6690	3880	58.0%	1/37	0/37	0/37
N +1000	3	4413	2800	63.4%	0/37	0/37	0/37

Abbreviations: EU = European Union, BSC = Best Standard Care, OAT = Optimized Asthma Therapy, PK = Pharmacokinetic, QL = Quality of Life, USA = United States of America.

## 4. Discussion

The results of the current study show that, in general, the proportion of women included in the CTs of omalizumab, benralizumab, reslizumab, mepolizumab and dupilumab in severe asthma was higher (60.4%) than the percentage of men. This percentage of females included in the studies is similar to the percentage of women with severe asthma reflecting a low gender bias regarding the inclusion of women in these CTs [67]. However, the separate analysis by sex of the main variable was carried out in only 5 of the 37 studies included, only 1 of the 37 trials discussed results separated by sex and no study included the concept of gender in the text. Additionally, the mean percentage of female authors among all the authors was low, just 17.5%.

Previous systematic reviews of gender bias that characterized women’s participation in HIV (human immunodeficiency virus) [68] or depression [10] clinical studies, concluded that this population was under-represented, so our work proves that the main CTs of the mAb used in severe asthma achieved, at least, a larger inclusion of women. Possible explanations for this fact could be that mAb are the most recent therapy for asthma, and consequently international recommendations [27–29] would have had an impact on the design of the CTs. However, the CTs included showed far-from-negligible gender bias in other variables such as sex-stratification of the main and secondary outcomes, the discussion of the results analyzed by sex and the absence of the concept of “gender” in the text. A potential reason why sex and gender considerations were not included is that sex and other demographic information such as age or race would have been analyzed as a covariate in some of these trials. However, guidelines recommend the inclusion of this valuable information in published studies [3–5]. Additionally, none of the trials followed a hormonal interaction approach to analyze the potential interaction with drugs such as hormonal contraceptives.

Several studies have proven that asthma affects men and women differently [17–19]. According to The Prevention and Incidence of Asthma and Mite Allergy (PIAMA) study [69], a unique study that recruited more than 4000 women and assessed more than 3300 of their children, exists a sex disparity in asthma: until puberty, asthma is more common and severe in boys, but after puberty, this disease becomes more common in women. Some recent articles have found an association between asthma and female sex hormones that could explain this fact [20]. Although the relationship remains unclear, the main hypothesis is that estrogen fluctuations directly modulate immune pathways crucial in asthma pathogenesis because of the anti-inflammatory action of these hormones [18]. In spite of all these reasons, none of the CTs analyzed the interaction between hormone replacement therapy and the study drug or investigate the effects of the phase of the menstrual cycle on the response to the drug. This fact goes against international recommendations and could respond to a possible attempt to avoid increases in the final cost of trials.

Furthermore, we found that more than half of the trials did not state whether pregnancy was a reason for exclusion. Even so, it is proved that there is a connection between pregnancy and asthma severity, but with a variable effect [70].

The CTs of the five mAb approved for the treatment of severe asthma were included in this study. Nevertheless, the percentage of women included in the CTs of the different drugs varied between them. Omalizumab’s trials were the ones that included a smaller proportion of women, probably related to the fact that it was the first drug launched and therefore its trials started earlier. However, in the two latest CTs of omalizumab, conducted both in 2016, an analysis by sex of the main outcome was performed. In contrast, the most recent CTs of benralizumab included the highest percentage of women, two of them analyzed the main outcome by sex and one discussed the results based on sex. On the other hand, the design of dupilumab’s or reslizumab’s CTs, was similar so the percentage of women included was almost identical. It could be explained by the presence of common research authors in these studies.

We should also mention that we included CTs carried out just in children, adults, or both. It is remarkable that a clinical study that enrolled more than 400 inner-city children, adolescents and young adults (6–20 years old) [58] was the only one that brought up socio-economical aspects from the participants, although the percentage of women was below the average. Besides, another publication included males and premenarchal females aged 6 to 12 years [49]. In this case, despite the interest in the effects of the phase of the menstrual cycle on the response to the drug, sex-related variables were not included in the design.

The main strength of this work is that it is the first systematic review performed on the recently commercialized mAb used in severe asthma which tries to assess gender of bias in CTs. Besides, two of the largest health databases that incorporate articles from the highest-impact medical journals, PubMed and EMBASE, were employed without date and language restrictions, and both CTs conducted in adults and children were included.

The main limitation was that the exclusion of post-hoc trials could prevent the inclusion of studies that subsequently evaluated variables based on sex. Moreover, pilot studies were excluded because our study assesses variables that are generally evaluated at the end of the clinical trial and preliminary results from pilot studies were commonly included in larger CTs. Similarly, short reports and letters to the editor were excluded due to the absence of complete data from CTs. The design of the study was limited to variables that were included in the CTs such as location or the phase of the study, but we did not analyze other relevant variables such as race or socioeconomic status. Therefore, we are awarded that evaluating the adequacy of women’s representation in CTs involves a more complex effort, so further studies should corroborate these results. Additionally, the cutoff for adult and pediatric age has been established at the age of 12, since most CTs distinguish between patients older or younger than 12 years. However, some adolescents may not reach puberty until they are not over the age of 12.

In conclusion, women represented more than half of the patients recruited in CTs of mAb for the treatment of severe asthma. The proportion of women in these CTs was higher than that reported by previous studies about other chronic diseases. However, the analysis of the main and secondary variables by sex or gender, as well as the discussion of the results separately by sex, are limited. Therefore, a potential gender bias in these CTs is found.

## Supporting information

S1 Checklist PRISMA 2009 checklist. (DOC)
